# An LSTM-Based Method with Attention Mechanism for Travel Time Prediction

**DOI:** 10.3390/s19040861

**Published:** 2019-02-19

**Authors:** Xiangdong Ran, Zhiguang Shan, Yufei Fang, Chuang Lin

**Affiliations:** 1 School of Computer Science and Technology, University of Science and Technology Beijing, Beijing 100083, China; fangyufei@sic.gov.cn; 2 Informatization and Industry Development Department, State Information Center, Beijing 100045, China; 3 College of Computer Science and Software Engineering, Shenzhen University, Shenzhen 518060, China; 4 Department of Computer Science and Technology, Tsinghua University, Beijing 100084, China chlin@tsinghua.edu.cn

**Keywords:** attention mechanism, recurrent neural networks, travel time prediction

## Abstract

Traffic prediction is based on modeling the complex non-linear spatiotemporal traffic dynamics in road network. In recent years, Long Short-Term Memory has been applied to traffic prediction, achieving better performance. The existing Long Short-Term Memory methods for traffic prediction have two drawbacks: they do not use the departure time through the links for traffic prediction, and the way of modeling long-term dependence in time series is not direct in terms of traffic prediction. Attention mechanism is implemented by constructing a neural network according to its task and has recently demonstrated success in a wide range of tasks. In this paper, we propose an Long Short-Term Memory-based method with attention mechanism for travel time prediction. We present the proposed model in a tree structure. The proposed model substitutes a tree structure with attention mechanism for the unfold way of standard Long Short-Term Memory to construct the depth of Long Short-Term Memory and modeling long-term dependence. The attention mechanism is over the output layer of each Long Short-Term Memory unit. The departure time is used as the aspect of the attention mechanism and the attention mechanism integrates departure time into the proposed model. We use AdaGrad method for training the proposed model. Based on the datasets provided by Highways England, the experimental results show that the proposed model can achieve better accuracy than the Long Short-Term Memory and other baseline methods. The case study suggests that the departure time is effectively employed by using attention mechanism.

## 1. Introduction

Traffic efficiency is important to travellers and is a key indicator of the level of traffic services, especially with the sharp increase in vehicles and the congestion in transfer in road networks. Traffic prediction can help travellers reasonably arrange in road network and improve traffic efficiency. Thus, traffic prediction is still a hot research topic of intelligent transportation systems (ITS). The evaluation indexes of traffic service level (e.g., queue length, severity of incident, traffic volume and average speed) assist travellers in making decisions pre-trip and en-route. Travel time is very intuitive and easily understood by travellers. Thus, travel time is very useful and widely accepted as an evaluation index, thus travel time prediction is considered in this paper.

Travel prediction is a research topic based on modeling the complex non-linear spatiotemporal traffic dynamics in road network [1]. Various traffic prediction methods such as support vector machine regression (SVR), linear regression (LR), k-nearest neighbor regression (k-NNR), autoregressive integrated moving average (ARIMA), and recurrent state-space neural network (SSNN) have been proposed in this area. CNN methods have been successfully applied to traffic prediction and have achieved better performance in recent years. These methods use the implicit correlations between near neighbors by convolution function [2,3,4]. Long Short-Term Memory(LSM NN)-based methods have been successfully applied to traffic prediction and have achieved better performance in recent years [5,6] because they have a better ability to model the traffic dynamics in road network as they can model long-term dependence in time series and extract features from traffic data with recurrent feedback.

The existing LSTM NNs for traffic prediction have two drawbacks: they do not make use of the departure time through the links for traffic prediction, and the way of model long-term dependence in time series is not direct in terms of traffic prediction. Travel time has a correlation with its departure time because traffic dynamics has some periodicity with departure time when traffic flow is not free. Thus, LSTM NN with departure time can provide better performance in predicting travel time than LSTM NN without departure time. The function of modeling long-term dependence in LSTM NNs is usually finished by extracting context information. It is well known that this method is excellent for nature language process because there are contextual structure in nature language. The data from traffic dynamics have no contextual structure. Thus, we assume that LSTM NN is less effective for traffic prediction. LSTM NN may be improved in traffic prediction because each data point in traffic dynamics may directly affect the predicted value.

Attention mechanism has recently demonstrated success in a wide range of tasks and is implemented by constructing a neural network according to the task. The attention mechanism can concentrate on the differences of input features to better extract features when different aspects are considered.

In this paper, we propose a LSTM NN with attention mechanism for travel time prediction (LSAM) that is over the output layers of LSTM NN. In the proposed LSTM NN, we use attention mechanism to construct the depth of LSTM NN and to model long-term dependence. The attention mechanism integrates departure time into the proposed model. The departure time is used as the aspect of the attention mechanism. We use AdaGrad method for optimizing the proposed model. Based on the datasets provided by Highways England, the experimental results show that the proposed model can achieve better accuracy than the LSTM NN and other baseline methods. The case study suggests that the departure time is effectively employed by using attention mechanism.

The contributions of this paper can be summarized as follows:

- We propose an LSTM-based method with attention mechanism for travel time prediction. In the proposed model, the traditional recurrent way to construct the depth of LSTM NN for modeling long-term dependence is substituted by an attention mechanism that is over the output layers of LSTM. The departure time is used as the aspect of the attention mechanism and is integrated into the proposed model.
- Experiments were performed based on the dataset provided by Highways England. The experimental results show that the proposed model can achieve better accuracy than the existing LSTM NN and other baseline methods.
- The case study suggest that the attention mechanism can effectively concentrate on the differences of input features for travel time prediction. The proposed model is feasible and effective.

The remainder of this paper is organized as follows. Section 2 reviews related works on traffic prediction. Section 3 describes the LSTM-based model with attention mechanism for travel time prediction. Section 4 compares Random Walk (RW), Seasonal Autoregressive Integrated Moving Average (SARIMA), Linear Regression (LR), k-Nearest Neighbor Regression (k-NNR), Support Vector Machine Regression (SVR), Recurrent State-space Neural Network (SSNN) and LSTM NN and discusses the experimental results. Finally the paper discusses the conclusion and future work.

## 2. Related Work

### 2.1. Data-Driven Models for Traffic Prediction

Given that enough data are available, data-driven models are more appropriate for traffic prediction, specifically neural network approaches [1]. The function (f) of data-driven approaches that relates the explicative variables with the target variable is usually determined by using statistical inference and machine learning techniques. In this paper, these traffic prediction methods fall into two categories: the methods considering departure time and the methods not considering departure time. The methods considering departure time are SARIMA and the LR. The methods that do not consider departure time are k-NNR, SVR and neural networks (NNs).

Seasonal Autoregressive Integrated Moving Average is a time-series analysis method for traffic prediction. ARMA is a combination of Autoregressive (AR) model and Moving Average (MA) model. ARMA is usually used for traffic prediction under stationary traffic dynamics where the mean, variance and auto-correlation are unchanged. ARIMA is a generalization of ARMA. When the traffic dynamic is non-stationarity, the initial differencing step of ARIMA is used one or more times to eliminate the non-stationarity.

Ahmed and Cook [7] proposed using ARIMA to predict short-term highway traffic flow. The experiments show that ARIMA (p, d, q) of order (0, 1, 3) can better model the given datasets. Many ARIMA-based models have been proposed for traffic prediction in the past decades. For example, ARIMA model of order (0, 1, 1) is proposed to reproduce all original time series. The experiments show that it is the most adequate model to reproduce all original time series [8].

If a seasonal component is added into ARIMA, we gain the structure denominated model called SARIMA. In [9], Williams et al. asserted that a one-week lagged first seasonal difference applied to discrete interval traffic data will yield a weakly stationary transformation. Resting on this assertion and the Wold decomposition theorem, Williams et al. presented a theoretical hypothesis that a univariate traffic data stream can be modeled as SARIMA. To validate the theoretical hypothesis, they performed experiments based on actual dataset from ITS and the empirical results are consistent with the theoretical hypothesis [9].

For traffic prediction, LR is one of the most typical non-parametric methods. Rice et al. discovered two naive predictors of travel time $TT_{e}\left(t+\delta \right)$: historical mean travel time $\mu_{TT}\left(t+\delta \right)$ and current status travel time $T_{e}^{⋆}\left(t\right)$ [10]. They concluded that travel time has a linear relation with its two naive predictors. Based on a dataset gathered from 116 single loop detectors along 48 miles in Los Angeles, they compared their method with other methods including principal components method and nearest neighbor method. The correctness of the conclusion is validated by the comparison.

In [11], k-NNR method is suggested as a candidate forecaster and is used for traffic prediction. The output value of k-NNR is the weighted average value of its k nearest neighbors on departure time. The empirical study describes the accuracy by comparing of the k-NN regression method to simple univariate time-series forecasts. In [12], k-NNR method is used to predict short-term traffic flow. In [13], k-NNR method is used to develop a model for dynamic multi-interval traffic volume prediction.

The idea of local weight is effective for traffic prediction. In [14], the effectiveness of local weight is validated. To suggest an approach for large-scale travel time prediction, Nikovski et al. presented an experimental comparison of several non-parametric methods [14], including LR, locally weighted regression, regression trees, k-NNR and neural networks. Although the non-linear methods have expected superiority over LR method, the locally weighted regression is the only non-linear method that can consistently outperform linear regression.

Given classification from the perspective of machine learning, the frequently used non-parametric methods usually fall into two categories: SVR and ANN.

The SVM method non-linearly maps input vectors to a very high dimension feature space, in which the linear decision surface for classification is constructed. SVM method does not depend on the dimension of input vectors. The high dimensionality space of SVM has high generalizability and big advantage for classification [15]. The SVR method is based on the SVM method. Thus, SVR-based methods are proposed for traffic prediction and achieve good performance. In [16], the SVR predictor with a radial basis function (RBF) kernel is proposed for travel time prediction. Based on the real highway traffic data, the proposed method achieves better performance than current-time predictor and historical-mean predictor. In [17], the incremental SVR method was proposed for traffic flow prediction. Based on the data sequentially collected by probe vehicles or loop detectors, the experimental results show that the proposed method is superior to the back-propagation neural network. In [18], an online version of SVR is proposed for short-term traffic flow prediction under atypical conditions (such as vehicular crashes, inclement weather, work zone and holidays).

Traffic prediction is based on modeling the complex non-linear spatiotemporal traffic dynamics in road network [1]. Many different types of neural networks have been proposed for traffic prediction including auto-encoders (AEs) [19], multi-layer perceptrons (MLP) [20] and recurrent neural networks [5,6,21].

In [19], Yisheng et al. rethought the traffic prediction problem based on deep architecture models, because existing traffic prediction methods are shallow and cannot live up to many real-world applications. Auto-Encoders (AEs) are proposed to learn generic traffic flow features and to predict traffic flow. AEs are trained on a greedy layer-wise way. Experimental results demonstrate that the proposed method for traffic flow prediction has superior performance compared to the baseline methods.

Polson et al. proposed a deep learning model for traffic flow prediction. The proposed model consists of a sequence of full-connection layers with activation function tanh to extract features. The proposed model is the same as MLP. The experimental data are from 21 road segments. The road segments span thirteen miles and are the major corridor that connects Chicago’s southwest suburbs to its central business district [20]. Based on the experimental data, the effectiveness of the proposed model is validated. In experiments, the amount of input nodes is the amount of road segments.

The topology of neural network should be derived from traffic-related consideration. Thus, Elman RNN [22] is referred to as SSNN in [1]. The topology of SSNN consists of input layer, hidden layer, output layer and context layer. The input layer receives traffic data (such as traffic flow and average speeds) on the main carriage way, on-ramps and off-ramps (if available). The output layer consists of one neuron that calculates the predicted travel time. The context layer stores the previous internal states of the model. The hidden layer receives inputs from input layer and then stores them in context layer, finally transforming them into output layer.

CNN methods may improve the predictive accuracy by transforming traffic into images and using the implicit correlations in the nearest neighbors [2,3,4]. In [2], a CNN method is proposed for large-scale, network-wide traffic speed prediction. In [3], a fusion of CNN and LSTM is proposed for short-term passenger demand prediction. In [4], a CNN method with an Error-feedback RNN is proposed for continuous traffic speed prediction.

In [6], LSTM NN is proposed for traffic prediction. Based on the traffic speed data from two microwave traffic detectors that are deployed along the express-way without signal controls, the effectiveness of LSTM NN for traffic prediction is validated. In [5], LSTM NN is proposed for travel time prediction. Evaluation experiments are made based on the travel time dataset provided by Highways England. The experimental results show that travel time prediction error is relatively smaller than the baseline methods and 7.0% is the approximate median of the mean relative error of 66 links.

In summary, various techniques are involved in traffic prediction to improve the performance of traffic prediction. These prediction methods are proposed and evaluated based on specific traffic data separately, thus it is difficult to say which method is definitely superior over other methods in every situation. Neural networks can better capture complex non-linear spatiotemporal relationship. Neural networks, especially LSTM NN, are promising for traffic prediction.

The existing LSTM NNs for traffic prediction have two drawbacks: they do not use the departure time through the links for traffic prediction, and the way of modeling long-term dependence in time series is not direct in terms of traffic prediction. Thus, we assume that the way of modeling long-term dependence in time series may be improved by using more direct access. Travel time is correlated with departure time because traffic dynamics have some periodicity with departure time when traffic flow is not free. Thus, LSTM NN for traffic prediction may be improved by efficiently using of departure time.

### 2.2. Attention Mechanism

Attention mechanism has recently succeeded in image classification [23], neural machine translation [24], multimedia recommendation [25] and many other tasks, because it can concentrate on the effective parts of features adaptively.

In the tasks for image classification, it is computationally expensive to apply convolutional neural networks (CNN) on large images because the computational cost of CNN scales linearly with the number of pixels of input images. To address the problem of enormous computation cost, a novel RNN model with attention is presented in [23]. The attention mechanism helps the proposed model to adaptively select a sequence of regions from image or video and only processes the selected regions at high resolution.

In the tasks for machine translation, attentional mechanism selectively focuses on the effective parts of input sentences during translating and improves the accuracy of machine translation. Luong et al. proposed two approaches of attention mechanism for machine translation: a global approach that always attends to all source words and a local approach that only considers a subset of the source words at a time [24].

In the task of multimedia recommendation, existing collaborative filtering systems (CF) ignore the implicitness in the users’ interactions with multimedia content. In [25], a two-layer attention mechanism is proposed to extract implicit feedback. The bottom layer adaptively selects the informative implicit feedbacks on component level. The upper layer adaptively selects the informative implicit feedbacks on item level. The selected implicit feedbacks are incorporated into the classic CF model with implicit feedback.

In this paper, the attention mechanism is proposed to address the two drawbacks of LSTM NN. The traditional recurrent way to construct the depth of LSTM NN is substituted by attention mechanism. The attention mechanism is over the output layers of LSTM NN to model long-term dependence. The departure time is used as the aspect of the attention mechanism and the attention mechanism is used to integrate departure time into the proposed model.

## 3. Methodology

### 3.1. LSTM NN for Travel Time Prediction

**Figure 1:**
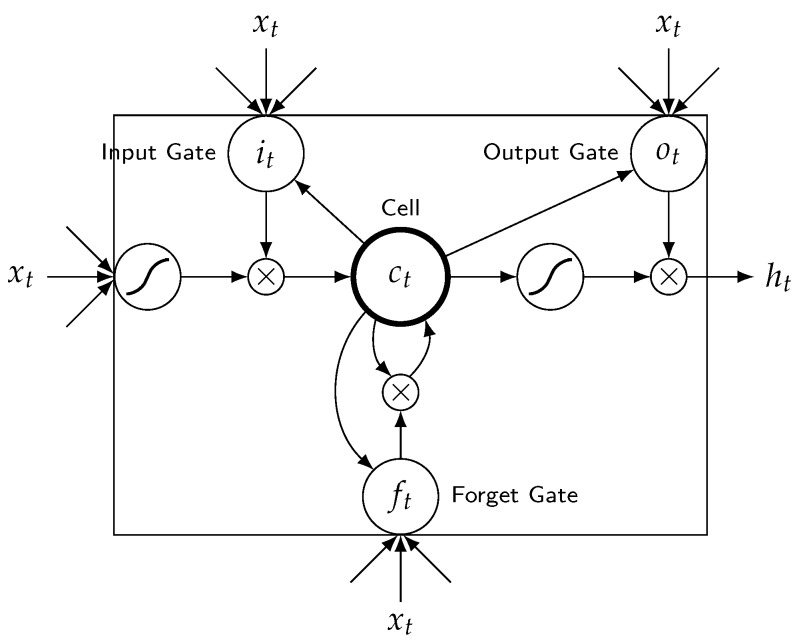
An architecture of LSTM unit.

**Figure 2:**
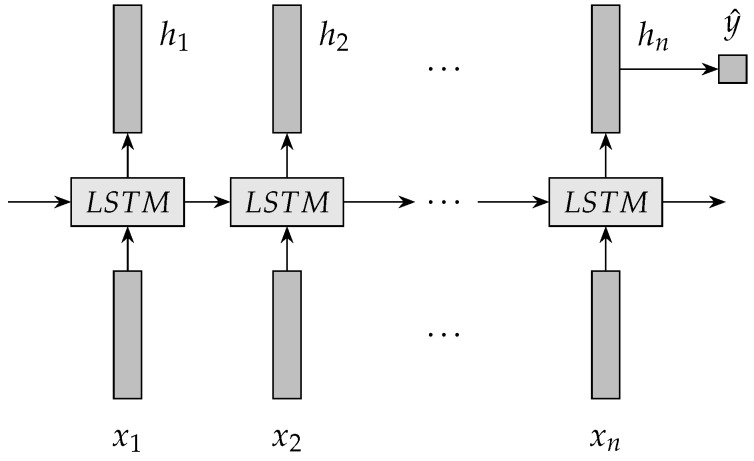
An architecture of LSTM NN for travel time prediction.

The transition function of standard RNN, $h_{t}=tanh\left(W\cdot \left[h_{t-1};x_{t}\right]+b\right)$, is a linear layer followed by a point-wise non-linear function (such as hyperbolic tangent function). Standard RNN suffers from a problem of vanishing or exploding gradients. The gradients may grow or decay exponentially over long sequences [26,27]. LSTM NN is a type of RNN with more sophisticated and powerful transition ability to process the gradients; it is proposed to address the problem. In the remainder of this section, we first review the architecture of LSTM unit illustrated in Figure 1. Then, we review the LSTM NN illustrated in Figure 2 and its usage for travel time prediction. LSTM unit has three gates and a memory cell: input gate $i_{t}$, forget gate $f_{t}$, output gate $o_{t}$, and memory cell $c_{t}$. These gates adaptively keep or override information in the memory cell, forget previous information and decide how to access memory cell [27].

The transition functions of LSTM unit are described in Equations (1)–(6). $W_{i},W_{f},W_{o}\in R^{d\times 2d}$ are the weight matrices of input gate, forget gate and output gate, respectively. $b_{i},b_{f},b_{o}\in R^{d}$ are the biases of input gate, forget gate and output gate, respectively. $W_{r}$ is the weight matrix of memory cell and $b_{r}$ is the bias of the memory cell. These parameters are learned during LSTM NN training. σ is a sigmoid function. ⊙ denotes element-wise multiplication. $x_{t}$ is the input vector that is fed into the input layer of the LSTM unit on step *t*. $h_{t-1}$ is the output vector of the hidden layer of the LSTM unit on step t−1 and is fed into the LSTM unit on step *t*. $h_{t}$ is the output vector of the hidden layer of the LSTM unit on step *t*. $c_{t-1}$ denotes the memory cell of the LSTM unit on step t−1. $c_{t}$ denotes the memory cell of the LSTM unit on step *t*.

(1) $$\begin{array}{cc} i_{t} & =\sigma (W_{i}\cdot \left[h_{t-1};x_{t}\right]+b_{i}) \end{array}$$

(2) $$\begin{array}{cc} f_{t} & =\sigma (W_{f}\cdot \left[h_{t-1};x_{t}\right]+b_{f}) \end{array}$$

(3) $$\begin{array}{cc} o_{t} & =\sigma (W_{o}\cdot \left[h_{t-1};x_{t}\right]+b_{o}) \end{array}$$

(4) $$\begin{array}{cc} g_{t} & =tanh(W_{r}\cdot \left[h_{t-1};w_{t}\right]+b_{r}) \end{array}$$

(5) $$\begin{array}{cc} c_{t} & =i_{t}⊙g_{t}+f_{t}⊙c_{t-1} \end{array}$$

(6) $$\begin{array}{cc} h_{t} & =o_{t}⊙tanh\left(c_{t}\right) \end{array}$$

In LSTM NN, LSTM units are placed end to end. LSTM units are used recursively to extract features from the complex non-linear spatiotemporal traffic data on road networks. LSTM NN recursively transits $x_{t}$, $h_{t-1}$ and $c_{t-1}$ into the output vector $h_{t}$. Finally, a vector $h_{n}$ is obtained that represents the predicted travel time.

An architecture of LSTM NN for travel time prediction is illustrated in Figure 2. {$x_{1},x_{2},\ldots ,x_{n}$} is an input sequence of LSTM NN. For travel time prediction, every item in the input sequence is a travel time value. LSTM NN uses the transition functions to extract {$h_{1},h_{2},\ldots ,h_{n}$} from the input sequence. {$h_{1},h_{2},\ldots ,h_{n}$} is the output vectors of the hidden layer of LSTM NN. $h_{n}$ represents the output value of LSTM NN. A predictor is usually linked to the end of LSTM NN $h_{n}$. A single layer neural network is usually used to implement the predictor. Based on the predictor, vector $h_{n}$ is transited into travel time $\hat{y}$ that is a real number. The predictor is formulated by Equation (7).

(7) $$\begin{array}{cc} \hat{y} & =W_{s}\cdot h_{n}+b_{s} \end{array}$$

### 3.2. Attention Mechanism

**Figure 3:**
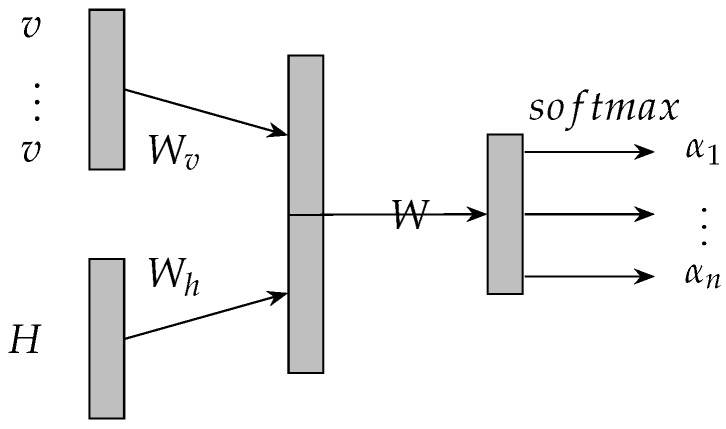
An architecture of attentive neural network.

Attention mechanism has recently demonstrated success in a wide range of tasks [23,24,25]. It is implemented by constructing a neural network according to the corresponding tasks. The neural network that is used for attention mechanism is usually called attentive neural network. An attentive neural network is illustrated in Figure 3. The transition functions of the attentive neural network are described by equations from Equations (8)–(10). *H* is an matrix and denotes the extracted features by prediction model, such as the aforementioned matrix $\left[h_{1},h_{2},\ldots ,h_{n}\right]$. $e_{n}\in R^{n}$ is a vector of 1 s and $v_{a}$ is the embedding of aspect for attention mechanism. α is a vector and denotes the attention weights to features *H*. *r* is the final output of the attentive neural network and denotes the weighted sum of features *H*.

(8) $$\begin{array}{cc} M & =tanh(\left[\begin{array}{c} W_{h}H\\ W_{v}v_{a}⊗e_{n} \end{array}\right] \end{array}$$

(9) $$\begin{array}{cc} \alpha & =softmax(w^{T}M) \end{array}$$

(10) $$\begin{array}{cc} r & =H\alpha^{T} \end{array}$$

Based on aspect, attention mechanism effectively extracts the features in input sequences. Specifically, attention mechanism allows the model to attend over features based on an aspect, thereby it mitigates the model bottleneck. The attention mechanism lets the model capture the whole traffic dynamics in input sequence. The attention mechanism only informs our proposed model to accumulate the parts of features needed to attend over while reading the input sequence and accumulating to a representation in the cell state.

### 3.3. LSTM-Based Method with Attention Mechanism

Based on the aforementioned discussion, we propose an LSTM-based model with attention mechanism for travel time prediction in this section. The proposed model better captures the relationship between history travel time and predicted travel time. First, the proposed model is a tree structure and is not a chain structure. The LSTM units are not placed end to end in the proposed model. The attention mechanism is used to construct the depth of LSTM NN and the structural pattern is different from the unfold way of standard LSTM NN. The attention mechanism is over the output layer of each LSTM unit to model long-term dependence. The proposed method models long-term dependence in time series by direct and efficient travel time prediction. Second, the proposed model uses the departure time through the links for traffic prediction. The proposed model integrates departure time for applying some periodicity with departure time. The departure time is used as the aspect of the attention mechanism. If departure time aspect is employed, the attention mechanism allows the proposed model to capture more important part of input sequences than LSTM NN without departure time.

**Figure 4:**
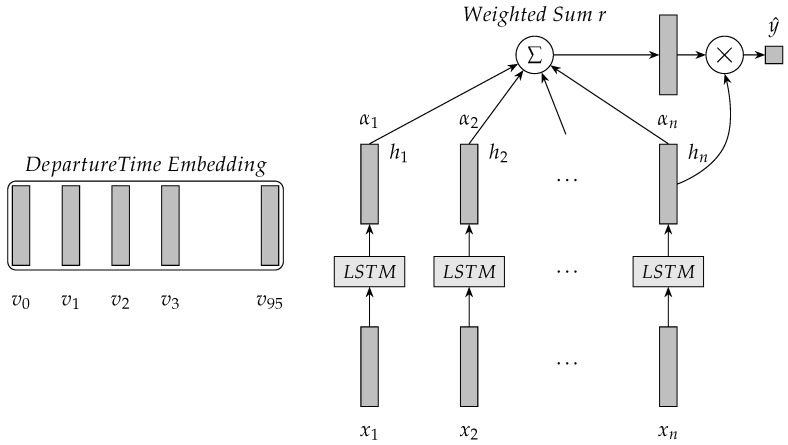
Architecture of the LSTM-based model with attention mechanism for travel time prediction.

The architecture of the LSTM-based model with attention mechanism for travel time prediction is illustrated in Figure 4. In Figure 4, $\left[x_{1},x_{2},\ldots ,x_{n}\right]$ denotes the input sequence into the proposed model. Let the departure time for the prediction be at 9:30 and *n* is 7, then the input sequence $\left[x_{1},x_{2},\ldots ,x_{n}\right]$ is at [7:45, 8:00, 8:15, 8:30, 8:45, 9:00, 9:15].

Based on the given dataset the time interval (time period) is 15 min. Thus, one day contains 96 time intervals and 96 departure times. In the proposed model, the randomly initialized 96 embeddings for the departure times are from 0 to 96. The embeddings are learned during model training. Let departure time be at 9:30, then the index of the departure time in the embeddings is 38 and $v_{3}8$ is selected as the departure time aspect of the attention mechanism. $v_{3}8$ is $v_{a}$ in Equation (8). $\left[h_{1},h_{2},\ldots ,h_{n}\right]$ in Figure 4 denotes *H* in Equation (8). $\left[a_{1},a_{2},\ldots ,a_{n}\right]$ in Figure 4 is *a* in Equation (9) and are attention weights on features *H*. *r* in Figure 4 is computed using Equation (10). The end of the proposed model is a predictor that is a single layer neural network. The predictor for travel time value refers to Equation (12).

(11) $$\begin{array}{cc} h_{s} & =tanh(W_{p}r+W_{x}h_{n}) \end{array}$$

(12) $$\begin{array}{cc} \hat{y} & =W_{s}h_{s}+b_{s} \end{array}$$

### 3.4. Models Training

We use a back-propagation method to train the proposed model. The back-propagation method is an end-to-end way method. The training is an optimization process to minimize the reward function (loss function) of the proposed model. In this paper, the reward function is an absolute loss function. We minimize the reward function value while the input sequences are sequentially fed into the proposed mode. The parameter set of the proposed model includes hyper-parameters, weight parameters and bias parameters. We choose the hyper-parameters of the proposed model on validation set, adjust the weights and biases of the proposed model on training set and evaluate the performance of the proposed model on test set. *y* is the observed travel time, $\hat{y}$ is the corresponding predicted travel time, *J* is the cost value, and *n* is the number of the travel time sequences.

(13) $$\begin{array}{cc} loss & =\underset{\Theta }{argmin}J\left(y,\hat{y}\right) \end{array}$$

(14) $$\begin{array}{cc} J(y,y_{pred}) & =\sum^{n}{\left(y-\hat{y}\right)}^{2} \end{array}$$

In our experiments, we uses a mini-batch gradient descent optimization method, AdaGrad [28], to training the proposed model. AdaGrad adapts its learning rate to the parameters of the proposed model. AdaGrad performs much larger updates for infrequent parameters than frequent parameters and is well suit to deal with sparse data. Dean et al. [29] found that AdaGrad greatly improves the robustness of SGD. The rules of AdaGrad to update parameters are described by Equations (15)–(17).

(15) $$\begin{array}{cc} \theta_{t+1,i} & =\theta_{t,i}-\frac{\eta }{\sqrt{G_{t,ii}}+\varepsilon }\cdot g_{t,i} \end{array}$$

(16) $$\begin{array}{cc} g_{t,i} & =\nabla_{\theta }J(\theta_{i}) \end{array}$$

(17) $$\begin{array}{cc} G_{t,ii} & =\sum^{t}g_{t,i}^{2} \end{array}$$

AdaGrad modifies the general learning rate of parameter $\theta_{i}$ at time step t+1 based on η and gradients $\theta_{i}$ and $G_{t}$ at time step *t*. Here, $G_{t}\in R^{d\times d}$ is a diagonal matrix. Each element on the diagonal line of matrix $G_{t,ii}$ is a squared sum over $g_{t,i}$. ε is a smoothing term that avoids division by zero (usually $1\times 10^{-8}$). Let learning rate η be 0.1. A main benefit of AdaGrad is that it eliminates the need to manually adjust the learning rate of model training [28].

Based on Figure 4 and its equations, the parameter set of the proposed model is $\left\{W_{i},b_{i},W_{f},b_{f},W_{o},b_{o},W_{c},b_{c},v,W_{h},W_{v},w,W_{p},W_{x},W_{s},b_{s}\right\}$. To accelerate training, a 4-G GPU computer with CUDA [30] was used for the proposed model. The initial size of CNMeM was enabled with 80.0% memory. Theano was used for implement our proposed model.

## 4. Experiment

We adopted three evaluation indices to evaluate the travel time prediction accuracy of the proposed model: the mean absolute error (MAE), the mean absolute percentage error (MAPE), and the RMS error (RMSE). The evaluation indices are described by Equations (18)–(20). *n* is the length of the given test set, *y* is the observed travel time and $\hat{y}$ is the predicted travel time.

(18) $$\begin{array}{cc} MAE & =\frac{1}{n}\sum_{i=1}^{n}\left|y_{i}-\hat{y}\right| \end{array}$$

(19) $$\begin{array}{cc} MAPE & =\frac{1}{n}\sum_{i=1}^{n}\frac{|y_{i}-\hat{y}|}{y_{i}} \end{array}$$

(20) $$\begin{array}{cc} RMSE & ={\left[\frac{1}{n}\sum_{i=1}^{n}{\left(y_{i}-\hat{y}\right)}^{2}\right]}^{\frac{1}{2}} \end{array}$$

### 4.1. Dataset

The experiments were performed on a dataset provided and managed by the Highways Agency in England [31]. This dataset provides average journey time (travel time) within 15-min time periods (time intervals) on all motorways and “A” roads. We selected a database from 1 March 2015 to 31 March 2015. The precision of travel time is 1 s. The dataset is described in Table 1, where one row is one time period.

The latest journey time and traffic data are available in [31]. This data series provides average journey time, speed and traffic flow information for 15-min periods since April 2009 on all morotways and “A” roads managed by the Highways Agency, known as the Strategic Road Network, in England. Journey times and speeds are estimated using a combination of sources, including Automatic Number Plate Recognition (ANPR) cameras, in-vehicle Global Positioning Systems (GPS) and inductive loops built into the road surface. Journey times are derived from real vehicle observations and imputed using adjacent time periods or the same time period on different days.

### 4.2. Task Definition

We predicted the travel time through links at time interval. Given one link selected, we predicted the travel time through the link at certain time interval. We divided the dataset that is related to the link into three parts: the data from 1 March to 21 March for the training set (approximately 87.1%), those on 28 March for the validation set (approximately 3.2%), and those from 29 March to 31 March for the test set (approximately 9.7%).

**Input sequences and its corresponding outputs:** The input sequences and its corresponding outputs were constructed from the database based on the selected link. The input sequences were fed into the proposed model and the corresponding outputs were observed values. The transition functions from the database to the input sequences are described in Equations (21) and (22).

(21) $$L=\left[\begin{array}{c} r_{1}\\ r_{2}\\ r_{3}\\ \cdots \\ r_{m} \end{array}\right]\overset{\;f\;}{\to }X=\left[\begin{array}{cc} \begin{array}{cccc} r_{1} & r_{2} & \cdots & r_{w}\\ r_{2} & r_{3} & \cdots & r_{w+1}\\ \cdots \\ \cdots & \cdots & \cdots & r_{m-1} \end{array} & \begin{array}{c} y_{1}\\ y_{2}\\ \cdots \\ y_{m-w} \end{array} \end{array}\right]$$

(22) $$y_{i}=r_{w+i}$$

Given link *L*, and *r* is the row in Table 1. Based on the task, The field average JT in Table 1 was selected as the item of input sequences. The values of average JTs were formatted into real numbers. Given the unfolded size of the proposed model is *w*, there are *w* LSTM units in the proposed model.

Matrix *X* represents the input sequences and its corresponding output for the proposed model and is generated from *L* by using Equations (21) and (22). Matrix *X* is fed into the the proposed model row by row for training or testing. For example, $\left[r_{1},r_{2},\ldots ,r_{w}\right]$ is regarded as an input sequence, and $y_{1}=r_{w+1}$ is regarded as the corresponding output.

There are 31 days in the selected database and 96 time periods per day (one time period is 15 min). Based on the selected database, there are m=2976(31×96) rows in matrix *L* and m−w rows in matrix *X*.

**Normalizing:** Normalization: Normalization is a very effective method to prepare input sequences. Our experiments showed that normalization of the dataset is important for the training process and can avoid overflows in calculation. In our experiments, the input sequences and its corresponding output were rescaled to the range of 0 to 1 because the activation functions in the gates of LSTM units are sigmoid functions.

### 4.3. Parameters Setting for the Proposed Method

We needed to determine the amount of LSTM units in the proposed model because of its recursion. The amount also decides the amount of item in one input sequence that is the *w* in Equation (21). In the proposed model, let the amount of LSTM units be *w* and the input sequence $\in R^{1\times w}$. Let the dimension of *h* and *o* of LSTM unit be 4. Hyper-parameters for the proposed model are described in Table 2.

**Figure 5:**
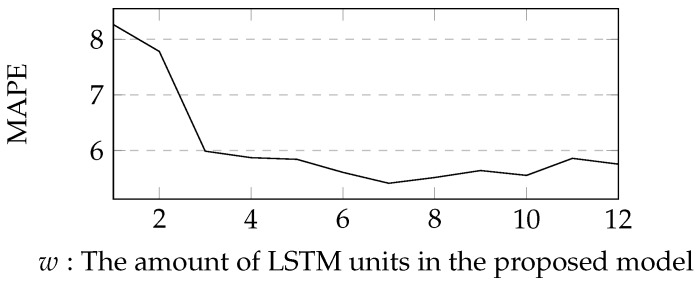
A change of MAPE when the amount of LSTM units in the proposed model increase. The experiment is made based on AL1896.

During validation process, we tried to obtain the optimal *w* of the proposed model. The definition domain of *w* ranges from 1 to 12. The experimental result is described in Figure 5. The optimal *w* was 7. The experimental result indicates that the amount of LSTM units should be sufficiently large to obtain a better accuracy. We obtained the hyper-parameters during validating process. The mini-batch size was 50. The initial learning rate of $W_{s}$$lr_{1}$ was 0.1, and the initial learning rate of other parameters $lr_{2}$ was 0.01.

$\left\{W_{i},b_{i},W_{f},b_{f},W_{o},b_{o},W_{c},b_{c},v,W_{h},W_{v},w,W_{p},W_{x},W_{s},b_{s}\right\}$ is the parameter set of the proposed model that is trained based on the given dataset. $\left\{W_{i},W_{f},W_{o},W_{c}\right\}\in R^{4\times 5}$ are the weight matrices of the input gate, forget gate, output gate and memory cell in the proposed model, respectively, which are described in Equations (1)–(6). $\left\{b_{i},b_{f},b_{o},b_{c}\right\}\in R^{4}$ are the respective biases described in Equations (1)–(6). $\left\{W_{h},W_{v}\right\}\in R^{4\times 4}$, $w\in R^{8\times 1}$ and $v\in R^{96\times 1}$ are the parameters for attention mechanism described in Equations (8) and (9). $\left\{W_{p},W_{x}\right\}\in R^{4\times 4}$ are the parameters in Equation (11). $W_{s}\in R^{4}$ and $b_{s}\in R^{1}$ are the parameters for the final predictor described in Equation (12).

### 4.4. Parameters Setting for the Baseline Methods

The baseline methods in this study were: RW, LR, k-NNR, SARIMA, SVR, SSNN, CNN and LSTM NN.

The simplest baseline method is RW method. In the RW method, the traffic conditions at time interval t+δ are equal to traffic conditions at time interval *t*. Thus, the function of the RW baseline method is: $X_{t+\delta }=X_{t}$, where *X* denotes travel time and *t* denotes time interval.

The LR method in our experiments is based on the work of Rice and Zwet [10]. In the literature, the learn rate of the parameters is set to 0.05. In our experiments, lag δ was set to 15 min and AdaGrad was used for the LR method. The parameter set of the LR method was {W1, W2, b1}. The function of the LR method was $X_{t+\delta }=W1\times X_{t}+W2\times \mu_{TT}\left(t+\delta \right)+b1$, where $\mu_{TT}\left(t+\delta \right)$ denotes historical mean travel time and is the historical mean of $X_{t+\delta }$ in the all previous days.

A k-NNR method was used for travel time prediction in our experiments. The output value of the k-NNR is a weighted average value of its k nearest neighbors. The time intervals of the k nearest neighbors are before the time intervals of the predicted value. In the k-NNR for travel time prediction, the ways of giving different weights to k nearest neighbors are usually uniform and distance. The uniform method is that the weight values are equal. The distance method is that the weight values are the inverse of their distance to the predicted value. We let the neighbors of k-NNR be 7 and all neighbors were previous to the predicted travel time on time intervals.

We built time series $\left\{X_{i}\right\}$ by using Equations (21) and (22). We presumed that $\left\{X_{i}\right\}$ is an SARIMA (p,d,q) (P,D,Q)${}_{S}$ process with period S. The SARIMA model was trained based on the literature [9]. The parameters of the SARIMA model in our experiments were set as follows: (p,d,q) was (1,0,1), (P,D,Q) was (0,1,1), and S was 96.

Linear kernel (linear), polynomial kernel (poly) and radical basis function kernel (rbf) were used as kernels of the SVR for travel time prediction in our experiments. The rbf kernel is proposed for traffic prediction in [16]. The independent term in poly and sigmoid kernel functions were set to 1e−3. The penalty parameter of the error term was set to 1.0. The degree of the polynomial kernel function was set to 3. $\frac{1}{\left|features\right|}$ was used as kernel coefficient for SVR. The tolerance for stopping criterion was set to 1e−3.

The SSNN in our experiments was trained based on the work in [1]. The parameters of the SSNN in our experiment are described as follows. The topology for the SSNN in our experiments was that the size of input layers, hidden layers and output layers are all 7. The recurrent connections were fixed at 1.0 and not subject to adjustment. The context units were initially set to 0.5. The function was $x_{c}\left(k\right)=x\left(k-1\right)$. The iteration time was 25. The size of the hidden layer was 4. The batch size was 25. The parameter set was $\left\{W_{f},b_{f},W_{s},b_{s}\right\}$, where $W\in R^{4\times 5}$ and $b\in R^{4}$. The $W_{f},b_{f}$ were for RNN and the $W_{s},b_{s}$ sdf for the final predictor that is the same as Equation (7).

The CNN in our experiments was $\left\{W,b,W_{1},W_{2},b_{2},W_{s},b_{s}\right\}$, where W,b is the convolution layer. The window of the convolution was 1×2. $W_{1}/inR^{30\times 10}$ and $Ws/inR^{10\times 1}$ was for the connection layers. The parameters of the final predictor was $W_{s},b_{s}$, which is the same as Equation (7). The iteration time was 40. The mini-batch was 25.

The LSTM NN was based on the work in [6]. The unfolded size of the LSTM NN was the same as the proposed model, i.e., 7. The iteration time was 25. The size of the hidden layer was 4. The batch size was 25. The parameter set was $\left\{W_{i},W_{o},W_{f},W_{c},b_{i},b_{o},b_{f},b_{c},W_{s},b_{s}\right\}$, as described in Equationss (1)–(7), where $W\in R^{4\times 5}$ and $b\in R^{4}$.

### 4.5. Similarity between the Prediction Value and the Observation Value

**Figure 6:**
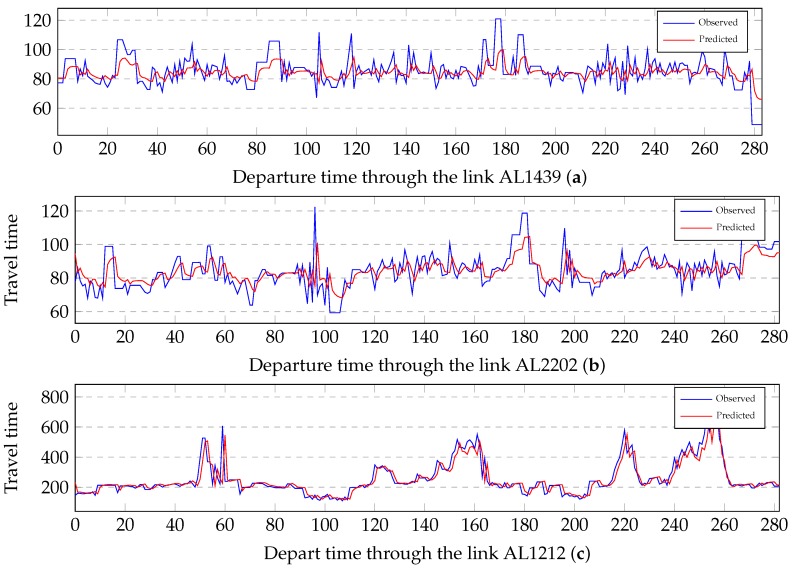
Comparisons between the predicted travel times and its observed travel times through the links under different traffic conditions: (**a**) a comparison under low traffic conditions; (**b**) a comparison under medium traffic conditions; and (**c**) a comparison under heavy traffic conditions.

The observed travel times and the corresponding predicted travel times through three short length links are illustrated to compare the similarity of change in Figure 6. Three links are under low (AL1439), medium (AL2202) and heavy (AL1212) traffic loads respectively. As shown in Figure 6, the predicted travel times exhibit similar change patterns of travel time with the observed travel times. The changes are in good agreement in low, medium and heavy traffic conditions.

**Figure 7:**
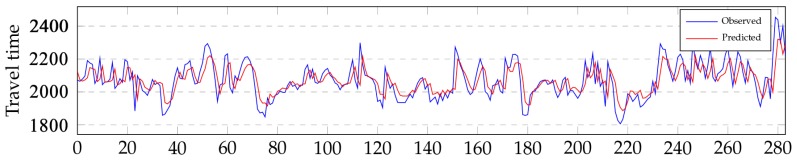
A comparison between the predicted travel times and the observed travel times through an medium length link L.

An medium length link L is illustrated in Figure 9. The observed travel times through link L are illustrated in Figure 7. The corresponding predicted travel times are also included in Figure 7 to compare the similarity of change. As shown in Figure 7, the predicted travel times exhibit similar change patterns of travel time with the observed travel times.

### 4.6. Accuracy Comparison Based on a Short Length Link

Based on link AL2202, the comparison between the proposed model and several baseline methods are described in this section. The length of AL2202 is 2.1 km.

**Prediction accuracy:** To evaluate the prediction accuracy of the proposed model, we compared the proposed model and the baseline methods based on short length link AL2202. The mean experimental results of these methods are given in Table 3. The experiment results show that the proposed model achieved better accuracy than the baseline methods. The proposed model outperformed the LSTM NN in terms of the accuracy and the proposed model also outperformed other baseline methods.

**Figure 8:**
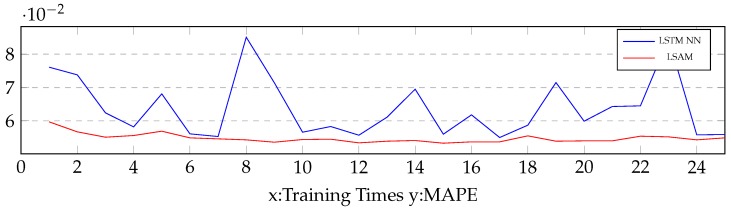
A comparison of convergence speed between the proposed model and the LSTM NN. The comparison is based on Link AL1896. The unfold size of the LSTM NN and the proposed model were 7.

**Comparison of convergence speed:** Based on the link AL1896, Figure 8 illustrates a comparison of the convergence speed between the proposed model and the LSTM NN. The result shows that the proposed model had faster convergence speed than the LSTM NN. Thus, the attention mechanism was sufficient to extract features from the input sequences and accumulate to a representation in memory cell. The attention mechanism informed the LSTM units to addend over the efficient parts of features.

### 4.7. Accuracy Comparison Based on a Medium Length Link

**Figure 9:**
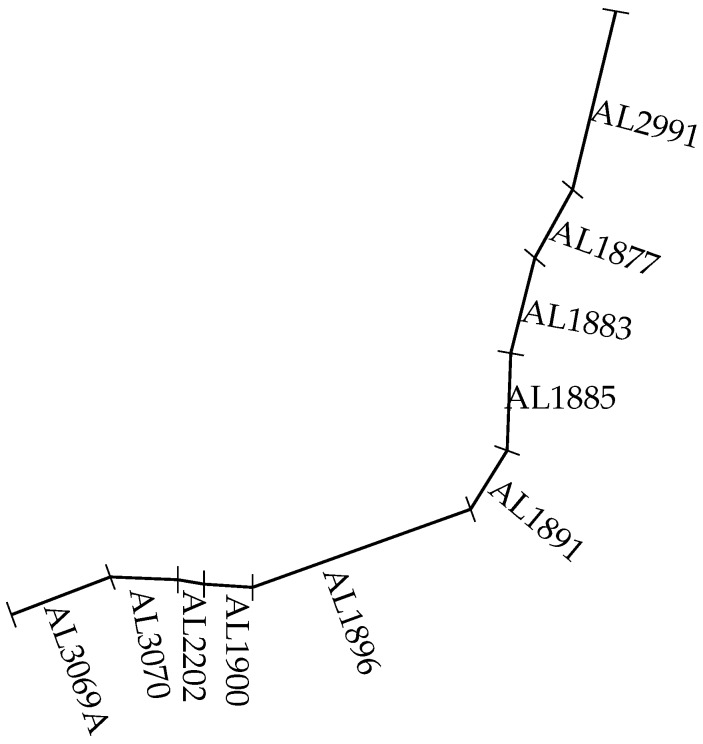
A road network consists of the links AL3069A, AL3070, AL2202, AL1900, AL1896, AL1891, AL1885, AL1883, AL1877 and AL2991 in Figure 9. The medium length link L consists of the links from link AL3070 to link AL 1877. A comparison between the proposed model and the proposed model was made based on link L.

We extended the comparison experiments based on an medium length link L on a road network. The tail link AL2291 and the head link AL3069A were removed from the road network to build a medium length Link L. Link L contained the links from AL3070 to AL1877, as described in Figure 9. The length of link L is approximately 54.38 km.

**Prediction Accuracy:** Based on link L, we compared the proposed model and the baseline methods for travel time prediction. The baseline methods and the corresponding setting are descried in the previous section. The comparable results is described in Table 4. The proposed model outperformed the LSTM NN and other baseline methods in terms of prediction accuracy. This conclusion based on the medium link L is consistent with the conclusion based on the shot length link AL2202.

**Figure 10:**
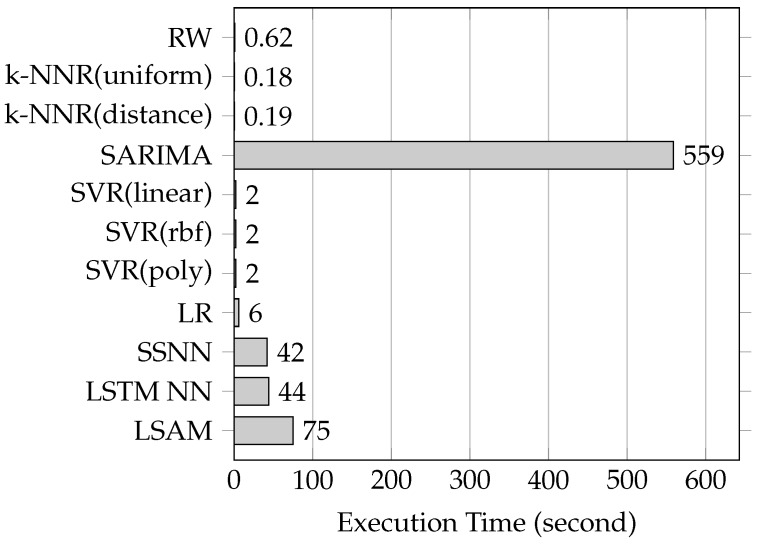
A comparison of time cost between the proposed model and the baseline methods.

**Time cost:** Based on medium length link L, we performed experiments to compare the time cost between the proposed model and the baseline methods. The experiments results are illustrated in Figure 10. The SARIMA method took the longest time to execute the training and testing. The methods based on NN (SSNN, LSTM NN and the proposed model) were likely to take more time training and testing than other none-NN methods (RW, k-NNR, SVR and LR) due to the complexity of the architecture and training of the NN-based methods. The time cost of the NN-based methods were within a reasonable boundary because of the rapid increase in the capacity of computers. The increase of the capability of computer promotes the recovery of NN-based methods, especially in the artificial intelligence domain.

### 4.8. A Further Evaluation on Link AL1167

The proposed model can be used for traffic prediction because there exist temporal relationship between the predicted travel time and the previous predicted travel time. To validate this assumption, we demonstrated the relationship by employing the Pearson correlation given by Equation (17), where *X* and *Y* denote two random variables with the same number of observations. The Pearson correlation was also used to explore the spatiotemporal correlations among the variables in short-term passenger demand prediction [3].

(23) $$\begin{array}{cc} Corr(X,Y) & =\frac{E(XY)-E(X)E(Y)}{\sqrt{E\left(X^{2}\right)-E^{2}\left(X\right)}\sqrt{\left(Y^{2}\right)-E^{2}\left(Y\right)}} \end{array}$$

We calculated the Pearson correlations between the predicted travel time and the previous predicted travel time. The predicted travel times was $\left\{T_{t}\right\}$ and the previous predicted travel times were $\left\{T_{t-1}\right\},\left\{T_{t-2}\right\},\left\{T_{t-3}\right\},\cdots ,\left\{T_{t-11}\right\}$, the time intervals of which are 1, 2, 3, ..., 11, respectively.

Figure 4 shows the correlations between $\left\{T_{t}\right\}$ and $\left\{T_{t-1}\right\},\left\{T_{t-2}\right\},\left\{T_{t-3}\right\},\cdots ,\left\{T_{t-11}\right\}$. It can be observed that the correlations between dropped gradually with the increase of time intervals. This means that there exited strong temporal correlations between the predicted travel times and its previous travel times. The correlations analysis validated the importance of the input sequences spanning time intervals and constructing the depth of LSTM to modeling long-term dependence.

**Figure 11:**
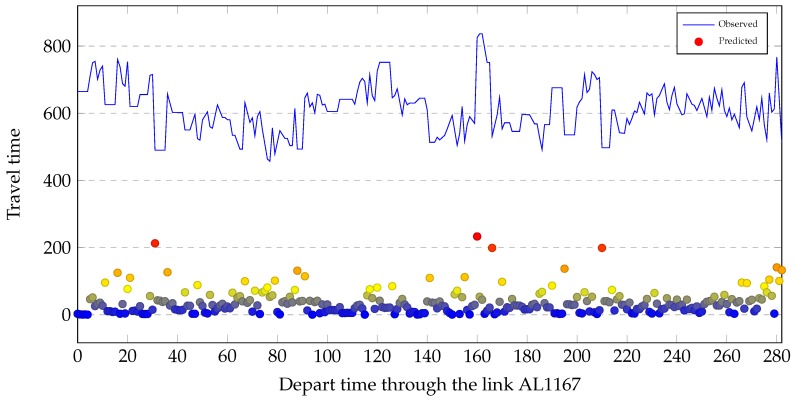
The residuals of the test set on link AL1996 over time. The horizontal axis is time interval and the vertical axis is travel time. The horizontal axis contains time intervals of three days and there are 96 time intervals per day.

To evaluate the effectiveness of the proposed model during periods of high volatility, we show the MAE (red dots) against the observed values (blue line) for three days on link AL1167 in Figure 11. We can see that the proposed model could capture the traffic regime changes, especially during high fluctuations, but the predictive power of the proposed model Was not uniform during the three days. There were four large errors in the 297 traffic predictions in Figure 11 when the traffic regime abruptly changed. The four large errors are marked in red. Nos. 1, 3 and 4 from left to right were observed at when traffic regime changed to congestion. No. 2 was observed when traffic regime changed to recovery.

**Figure 12:**
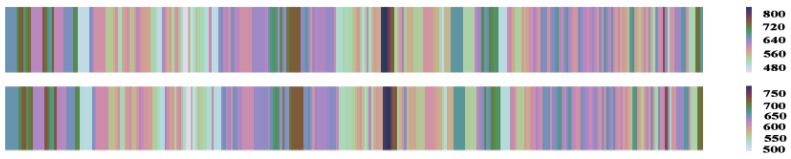
Heat plots of travel times during three days. The horizontal axis are time intervals that spans three days and 96 time intervals per day. The color depth expresses the travel time value on link AL1996.

To evaluate the predictive power of the proposed method to capture the traffic condition propagation in terms of travel time, we describe the travel time prediction results by using the heat maps on link AL1996 in Figure 12. The observed values are shown in the first plot and the predicted values are shown in the second plot. In contrast, we see that the proposed method properly captured both forward and backward congestion propagation during the three days.

**Figure 13:**
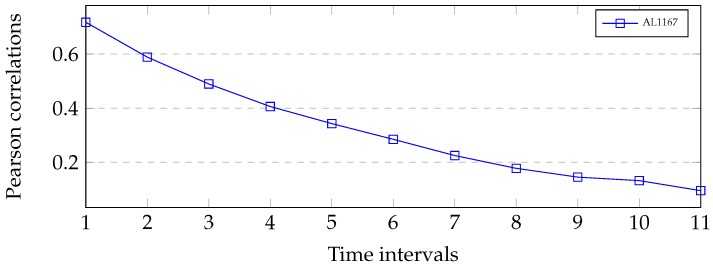
Travel time correlations on link AL1167 with length of 18.48 km. The best results in the table appear in bold.

Table 5 demonstrates the prediction performance of different methods with various time lags for AL1167. The method with the best performance is marked in bold. Based on AL1167, a general trend can be found that the prediction accuracies for the methods increased as the time lag became longer. We can see that the proposed model achieved the best prediction accuracy when time lag was 1. This conclusion is the same as the correlations in Figure 13.

### 4.9. Result Analysis

It is well known that the prediction errors of models can be decomposed into two main subcomponents: error due to “bias” and “variance”. The bias errors are the difference between prediction values and observed values. The bias errors are due to erroneous assumptions in the prediction model. High bias implicates under fitting that the models missed some relevant relations between features and predictive outputs. The variance error is a difference of bias error. The variance error is due to the sensitivity of model in the small fluctuations in training set. High variance implicates over fitting that the model transits the random noise in training data, rather than the intended outputs. There is usually a trade off between bias and variance [32].

**Bias discussion:** The proposed method outperformed all baseline methods in terms of bias. Thus, there was no under-fitting problem in the proposed model.

Traffic dynamic has some periodicity with departure time when traffic flow is not free. Thus, departure time was an effective input for traffic prediction. If the same input sequences were fed into LSTM NN, LSTM NN without departure time outputed the same predicted value even if the predicted values and their departure times were different. Obviously, LSTM NN without departure time was less effective for travel time prediction than the proposed model. The experiment results prove the hypothesis. Table 3 and Table 4 show that the proposed model has the smallest bias. The proposed model exhibited the best accuracy and can effectively predict travel time through the link. Specifically, using the departure time through the links in the proposed model was feasible and effective for improving LSTM NN for traffic time prediction. In the proposed model, departure time is used as the aspect of the attention mechanism and the attention mechanism is used to integrate departure time into the proposed model.

In the proposed model, the attention mechanism substitutes the traditional recurrent way to construct the depth of LSTM and model long-term dependence in time series. The new way is direct in terms of traffic prediction. The proposed model obtained the best performance and outperformed all other methods in terms of prediction accuracy. Thus, the new way is efficient to predict travel time through links.

Because link L is longer than link AL2202, the bias of the proposed model in Table 4 is smaller than it is in Table 3. This conclusion is the same as the conclusion in [16].

**Variance Discussion:**Table 4 shows that the variances of the proposed model is on the same order of magnitude as the baseline methods except the k-NN with uniform function and the SARIMA method. The proposed method outperformed seven of the baseline methods in terms of variance. Thus, there was no over-fitting problem in the proposed model.

### 4.10. Case Study

To validate the effectiveness of applying departure time and the new way to construct the depth of the proposed method, the experiments were repeated twice based on link AL2202. Let 10:00 be the departure time through link AL2202. The departure time of 10:00 was chosen at random. The predicted travel times and their attention weights are recorded in Table 6 and Table 7.

**Method stability:** We compared the prediction accuracy MAPE of the proposed method between Table 6 and Table 7. The results on 29 March indicate that the prediction accuracy was not affected by the training process. Thus, the proposed method was stable. We came to the same conclusion on 30 and 31 March.

**The prediction of travel time change tendency:** We compared the travel time at 10:00 with those at 9:45 on 29 March (Table 6); the observed travel time at 10:00 and the predicted travel time at 10:00 were shorter than the observed travel time at 9:45. In conclusion, the predicted travel time at 10:00 and the observed travel time at 10:00 had the same decreasing trend in their change.

We compared the travel time at 10:00 and those at 9:45 on 30 March (Table 6); the observed travel time at 10:00 and the predicted time at 10:00 were longer that the observed travel time at 9:45. In conclusion, the predicted travel time at 10:00 and the observed travel time at 10:00 had the same increasing trend in their change.

We compared the travel time at 10:00 and those at 9:45 on 31 March (Table 6); the predicted travel time at 10:00 and its observed travel time did not show the same change trend. The observed travel time at 10:00 was longer than the travel time at 9:45 and the predicted travel time at 10:00 was shorter than its travel time at 9:45. Perhaps, the proposed model could regress toward the mean, because the predicted travel time was too long.

The change trend of the predicted travel time or the regression toward the mean presented in Table 7 was the same as those in Table 6.

**Efficiency of the departure time aspect:**Table 6 and Table 7 show that the attention mechanism focused on the output features of the proposed model given a departure time. In the tables, the attention weights indicate the importance degree of the output features: the larger is the weight, the more important is the feature. If the predicted travel time were larger than its corresponding one-step travel time, the attention weights would be more focused on the the larger travel time. If the predicted travel time were smaller than the corresponding one-step travel time, the attention weights would be more focused on the small travel time. In conclusion, the attention mechanism could effectively focus on the output features to produce the weighted sum *r* in Figure 4 when dynamically given a departure time. It is an effective new way to construct the depth of the proposed method.

## 5. Conclusions

In this paper, we propose an LSTM-based method with attention mechanism for travel time prediction. We use the attention mechanism to construct the depth of the proposed model. The departure time is used as the aspect of the attention mechanism and is integrated into the proposed model. Comparison experiments were performed based on the database provided by Highways England. The experimental results show that the proposed model obtained better accuracy than the baseline model. The case study suggests that the attention mechanism can effectively concentrate on the differences of input features. Thus, the proposed model is feasible and effective.

As future work, we will improve the trend and avoid regression toward the mean. A potential direction would be the application of a local optimization approach.

## Figures and Tables

**Table 1:** Data description of the database provided by the Highways Agency in England.

Link Ref	Date	Time Period (0–95)	Average JT (s)	Link Length (km)
AL1896	1 March 2014	0	626.12	18.32
AL1896	1 March 2014	1	612.60	18.32
AL1896	1 March 2014	2	604.23	18.32
…	…	…	…	…

**Table 2:** Hyper-parameters for the proposed model.

Variable	Description	Value
*w*	The unfold size of the proposed model	7
*d*	The dimension of hidden layers and output layers of LSTM	4
mi	The mini-batch size	50
$lr_{1}$	The initial learning rate of $W_{s}$	0.1
$lr_{2}$	The initial learning rate of other parameters	0.01
norm	The range of the dataset normalization	(0, 1)

**Table 3:** A comparison between the proposed model and the baseline methods based on short length link AL2202. The best results in the table appear in bold.

Models		Departure Time in 15 min		
		RMSE	MAE	MAPE (%)
RW		9.787	2.5648	8.87
k-NNR	uniform	9.800	2.566	8.73
	distance	9.800	2.566	8.73
SARIMA		8.637	2.582	9.01
SVR	linear	8.202	2.487	8.30
	rbf	8.180	2.486	8.30
	poly	8.216	2.497	8.36
LR		10.323	2.786	8.92
CNN		9.270	2.716	8.41
SSNN		9.205	2.650	8.37
LSTM NN		8.472	2.485	7.34
LSAM		**8.133**	**2.356**	**7.01**

**Table 4:** A accuracy comparison between the proposed model and the baseline methods based on medium length link L. The best results in the table appear in bold.

Models		15-min on Training Set			15-min on Test Set			Variance (%)
		RMSE	MAE	MAPE (%)	RMSE	MAE	MAPE (%)	
RW		87.499	8.018	3.33	78.7770	7.5445	2.99	0.34
k-NNR	uniform	77.955	7.645	3.03	78.854	7.545	2.99	0.04
	distance	14.313	1.663	0.14	78.854	7.545	2.99	2.85
SARIMA		413.931	19.963	20.51	93.271	8.508	3.85	16.66
SVR	linear	80.628	7.778	3.14	72.983	7.408	2.90	0.24
	rbf	80.646	7.775	3.14	73.036	7.427	2.92	0.22
	poly	84.016	7.892	3.25	74.670	7.571	3.04	0.21
LR		83.963	7.897	2.96	92.880	8.565	3.45	0.49
CNN		83.093	7.878	3.20	72.131	7.399	2.87	0.33
SSNN		83.631	7.873	3.18	72.060	7.361	2.84	0.34
LSTM NN		80.885	7.781	3.14	71.631	7.318	2.83	0.31
LSAM		79.498	7.650	2.81	70.146	7.188	**2.69**	0.22

**Table 5:** The results of the proposed method with various time lag based on AL1167.

Models	Time Lag 1		Time Lag 2		Time Lag 3		Time Lag 4	
	MAE	MAPE	MAE	MAPE	MAE	MAPE	MAE	MAPE
CNN	6.432	6.87	6.947	7.92	7.080	8.28	7.267	8.75
SSNN	6.338	6.10	6.912	7.87	7.265	8.70	7.415	9.03
LSTM NN	5.979	5.95	6.858	7.78	7.197	8.47	7.342	8.98
LSAM	**5.788**	**5.61**	6.796	7.66	7.069	8.28	7.294	8.76

**Table 6:** Three experimental results collected in the first experiment of the proposed model.

Departure Time		8:15	8:30	8:45	9:00	9:15	9:30	9:45	10:00		
Steps Ahead		7-Step	6-Step	5-Step	4-Step	3-Step	2-Step	1-Step	Observed	Predicted	MAPE (%)
03-29	AverageJT	70.82	71.82	79.10	78.77	83.30	83.30	83.30	74.32	82.77	11.4
	Weights	0.227	0.222	0.142	0.133	0.096	0.090	0.089		
03-30	AverageJT	86.38	86.91	91.71	77.73	79.47	89.04	83.73	96.87	84.80	12.5
	Weights	0.148	0.118	0.083	0.199	0.203	0.109	0.140		
03-31	AverageJT	83.63	85.33	84.57	89.38	87.10	91.99	95.78	97.21	91.05	6.3
	Weights	0.222	0.170	0.170	0.124	0.137	0.100	0.076		

**Table 7:** Three experiment results collected in the second experiment of the proposed model.

Time Period		8:15	8:30	8:45	9:00	9:15	9:30	9:45	10:00		
Steps Ahead		7-Step	6-Step	5-Step	4-Step	3-Step	2-Step	1-Step	Observed	Predicted	MAPE (%)
03-29	AverageJT	70.82	71.82	79.10	78.77	83.30	83.30	83.30	74.32	82.80	11.4
	Weights	0.181	0.179	0.140	0.140	0.120	0.120	0.120		
03-30	AverageJT	86.38	86.91	91.71	77.73	79.47	89.04	83.73	96.87	84.54	12.7
	Weights	0.134	0.121	0.104	0.189	0.180	0.124	0.147		
03-31	AverageJT	83.63	85.33	84.57	89.38	87.10	91.99	95.78	97.21	90.81	6.6
	weights	0.170	0.150	0.161	0.137	0.150	0.125	0.107		
